# Effectiveness of Medical Nutrition Therapy in the Management of Patients with Obesity and Endometriosis: from the Mediterranean Diet To the Ketogenic Diet, Through Supplementation. The Role of the Nutritionist in Clinical Management

**DOI:** 10.1007/s13679-025-00662-8

**Published:** 2025-09-08

**Authors:** Luigi Barrea, Ludovica Verde, Giuseppe Annunziata, Peter Chedraui, Felice Petraglia, Gabriela Cucalón, Elisabetta Camajani, Massimiliano Caprio, Stefania Gorini, Giuseppe Gabriele Iorio, Raffaella Di Girolamo, Luigi Carbone, Sebastian Chapela, Evelyn Frias-Toral, Giovanna Muscogiuri

**Affiliations:** 1 Dipartimento di Psicologia e Scienze della Salute, Centro Direzionale, Università Telematica Pegaso, Naples, Via Porzio, Isola F2, 80143 Italy; 2 Department of Public Health, University of Naples Federico II, Naples, Via Sergio Pansini 5, Italy; 3 Department of Medicine, Division of Endocrinology, University of Arizona, Tucson, AZ USA; 4 Facoltà di Scienze Umane, della Formazione e dello Sport, Università Telematica Pegaso, Naples, Via Porzio, Centro Direzionale, Isola F2, 80143 Italy; 5 Escuela de Postgrado en Salud, Universidad Espíritu Santo, Samborondón, Ecuador; 6 Department of Experimental, Clinical and Biomedical Sciences Mario Serio, Department of Maternal and Child Health, University of Florence, Careggi University Hospital, Florence, Italy; 7 Escuela Superior Politécnica del Litoral, ESPOL, Lifescience Faculty, ESPOL Polytechnic University, Guayaquil, Campus Gustavo Galindo Km. 30.5 Vía Perimetral, P.O. Box 09-01-5863, Ecuador; 8 Laboratory of Cardiovascular Endocrinology, IRCCS San Raffaele, Rome, Italy; 9 Department for the Promotion of Human Sciences and Quality of Life, San Raffaele Roma Open University, Rome, Via di Val Cannuta 247, 00166 Italy; 10 Department of Neuroscience, Reproductive Science and Odontostomatology, University of Naples Federico II, Naples, Italy; 11 Department of Public Health, School of Medicine and Surgery, University of Naples Federico II, Naples, Italy; 12 Departamento de Bioquímica, Facultad de Ciencias Médicas, Universidad de Buenos Aires, Ciudad Autónoma de Buenos Aires, C1121ABE Argentina; 13 Equipo de Soporte Nutricional, Hospital Británico de Buenos Aires, Ciudad Autónoma de Buenos Aires, C1280AEB Argentina; 14 School of Medicine, Universidad Católica de Santiago de Guayaquil, Guayaquil, Av. Pdte. Carlos Julio Arosemena Tola, 090615 Ecuador; 15 Dipartimento di Medicina Clinica e Chirurgia, Unità di Endocrinologia, Diabetologia, Andrologia e Nutrizione, Università degli Studi di Napoli Federico II, Naples, Italy; 16 Dipartimento di Endocrinologia, Diabetologia, Andrologia e Nutrizione, Centro Italiano per la cura e il Benessere del Paziente con Obesità (C.I.B.O), AOU Federico II, Naples, Via Sergio Pansini 5, 80131 Italy; 17 Cattedra Unesco Educazione Alla Salute e Allo Sviluppo Sostenibile, University Federico II, Naples, Italy

## Abstract

**Purpose of the Review:**

This review aimed to summarize current evidence on the effectiveness of medical nutrition therapy (MNT) in the management of obesity and endometriosis, with a focus on dietary patterns such as the Mediterranean and Ketogenic diets, as well as nutritional supplementation. Additionally, it highlights the central role of the clinical nutritionist in implementing individualized, evidence-based interventions within multidisciplinary care.

**Recent Findings:**

Although the literature reports the existence of an inverse relationship between risk of endometriosis and body mass index, clinical evidence jointly reports that a condition of obesity is associated with greater disease severity. This, therefore, implies the need to identify the different phenotypes of patients with endometriosis at which to target a precision MNT.

Several dietary patterns and supplements have been investigated for their role in endometriosis management. The Mediterranean diet—rich in anti-inflammatory nutrients, fiber, and antioxidants—has been associated with decreased pain and improved quality of life. More recently, ketogenic diets have shown potential in modulating insulin signaling and inflammatory pathways, though clinical evidence remains limited. Supplementation with omega-3 fatty acids, N-acetylcysteine, resveratrol, vitamins C and E, and probiotics has demonstrated promising anti-inflammatory and antioxidative effects in both preclinical and clinical studies. Furthermore, attention is being directed toward the gut microbiota and its interaction with the immune and endocrine systems in women with endometriosis.

**Summary:**

Endometriosis is a chronic gynecological condition characterized by ectopic endometrial tissue, estrogen dependence, and persistent inflammation. It affects approximately 10% of women of reproductive age and is associated with pelvic pain, infertility, and reduced quality of life. While conventional treatment focuses on hormonal therapy and surgery, MNT is emerging as a non-invasive, supportive approach. Nutritional interventions can target key pathophysiological mechanisms of endometriosis, such as systemic inflammation, oxidative stress, and hormonal imbalance, offering potential symptom relief and improved clinical outcomes.

**Supplementary Information:**

The online version contains supplementary material available at 10.1007/s13679-025-00662-8.

## Introduction

Endometriosis is a chronic, estrogen-dependent, and inflammatory gynecological disorder characterized by the ectopic growth of endometrial-like tissue outside the uterine cavity [1, 2]. Affecting an estimated 5–10% of women of reproductive age worldwide, it presents with a constellation of debilitating symptoms, including dysmenorrhea, chronic pelvic pain, dyspareunia, and infertility [3]. Despite its high prevalence and significant impact on quality of life, the pathogenesis of endometriosis remains incompletely understood, involving a complicated combination of hormonal, immunological, genetic, and environmental factors [3].

Among the environmental factors, lifestyle plays a primary role. In particular, nutritional status, and more specifically body mass index (BMI) and body composition, are in a central position in the context of this relationship. In this scenario, the literature has historically reported the existence of an inverse relationship between endometriosis risk and BMI, with some observational studies suggesting a lower incidence among women with higher BMI, without implying a causal or protective effect of obesity. More recent evidence, however, has clarified the role of obesity in the progression of endometriosis, identifying this condition as predisposing to more severe disease [4]. This evidence implies the need to identify the different phenotypes of patients with endometriosis with normal weight or obesity in order to implement personalised treatment strategies.

Despite decades of research, current treatments for endometriosis remain limited, focusing mainly on symptomatic pain relief, hormonal therapy, or surgical removal of lesions—none of which effectively prevent recurrence [1]. These conventional strategies often come with drawbacks such as side effects, recurrence, or restricted use, underscoring the need for complementary, non-pharmacological alternatives [1].

In recent years, nutrition has garnered increasing interest as a modifiable lifestyle factor that may influence the development, progression, and symptomatology of endometriosis [5]. The rationale for incorporating nutrition into the clinical management of endometriosis stems from its ability to modulate key mechanisms implicated in disease pathophysiology: chronic systemic inflammation, oxidative stress, estrogen metabolism, and immune dysfunction [5]. Medical Nutrition Therapy (MNT), defined as a personalized, evidence-based approach to nutritional care provided by a trained clinician (typically a registered dietitian or nutritionist) [6, 7], offers a structured framework for addressing these pathophysiological pathways through dietary modifications and targeted supplementation.

Among the various dietary models explored, the Mediterranean diet (MedDiet) has emerged as one of the most promising in the context of endometriosis [8]. Characterized by high intake of vegetables, fruits, whole grains, legumes, nuts, fish, and extra virgin olive oil, along with limited consumption of red and processed meats and refined sugars, the MedDiet exerts well-established anti-inflammatory and antioxidant effects [9]. Its rich content of omega-3 polyunsaturated fatty acids, polyphenols, and fiber contributes to the modulation of prostaglandin synthesis, estrogen clearance via the gut microbiota, and systemic oxidative balance [9].

More recently, attention has turned to the potential application of the ketogenic diets (KDs) in endometriosis management [10]. Originally developed for the treatment of refractory epilepsy, the KD is a high-fat, low-carbohydrate diet that induces a state of physiological ketosis. This metabolic shift exerts notable anti-inflammatory effects [11, 12], which are particularly relevant in the context of endometriosis, a condition characterized by chronic inflammation [13]. By reducing systemic inflammation, the KD may help alleviate pelvic pain and other inflammatory symptoms associated with the disease [11–13]. Additionally, the KD lowers insulin levels and may influence estrogen biosynthesis by modulating insulin-like growth factor (IGF)−1 signaling and aromatase activity [11, 12]. While clinical evidence remains scarce, preliminary findings suggest that the KD may offer therapeutic benefits in women with endometriosis, especially those with comorbidities such as obesity or insulin resistance—conditions that not only worsen inflammatory status but are also known to exacerbate endometriosis symptoms [13].

Beyond dietary patterns, specific nutrients and supplements have shown therapeutic potential in mitigating the inflammatory and oxidative milieu of endometriosis [14, 15]. Notable examples include omega-3 fatty acids, known to compete with arachidonic acid and reduce the synthesis of pro-inflammatory prostaglandins; N-acetylcysteine (NAC), which acts as a precursor to glutathione and modulates cytokines expression; and antioxidants such as vitamins C and E, which reduce oxidative damage in peritoneal fluid [16]. Additionally, emerging evidence highlights the importance of gut microbiota in the immune-endocrine regulation of endometriosis, suggesting a potential role for prebiotics, probiotics, and dietary fiber in restoring microbial homeostasis and improving clinical outcomes [17].

Despite the promising role of nutrition in endometriosis management, there is still a lack of specific clinical guidelines for implementing MNT, as well as limited high-quality evidence supporting the efficacy of particular dietary patterns or nutritional supplements.

Based on this background, this review aimed to critically examine the current evidence on the role of MNT in endometriosis, focusing on the MedDiet and the KD, and targeted supplementation. Particular emphasis was placed on the clinical relevance of these strategies and the central role of the Nutritionist in translating scientific evidence into practical, personalized care.

## ENDOMETRIOSIS: the Point of View of the Gynecologist and the Endocrinologist

### Pathophysiology

Endometriosis is a benign disease characterized by the presence of endometrium-like tissue outside the uterus, affecting women of reproductive age. The most common symptoms of endometriosis are dysmenorrhea, dyspareunia, dysuria and dyschezia, and infertility [18].

The prevalence ranges between 2 and 10% of women of reproductive age and between 30 and 50% among infertile patients. There are several classifications, but the most current is based on the location of the lesions: ovarian endometrioma (OMA) (typical chocolate cysts), superficial peritoneal endometriosis (SUP), and deep infiltrating endometriosis (DIE) (deeper than 5 mm under the peritoneum). Extraperitoneal locations are also described (lung, diaphragm, or umbilicus), although with a reduced incidence. Of high interest is the observation that 30%−40% of endometriosis cases are associated with adenomyosis (infiltration by endometrial stroma and glands into the myometrium) [19, 20].

The pathophysiology of endometriosis is complex and implies multiple steps. Retrograde menstruation, vascular and lymphatic spread, and/or metaplasia/stem cells are the mechanisms, that drive eutopic endometrial cells to the ectopic locations. The retrograde menstruation hypothesis is the most accepted, implying the migration of endometrial fragments through the fallopian tubes to the peritoneum. This event occurs every month, and in most women, a scavenger system of autophagy/apoptosis eliminates these endometrial cells. When this mechanism does not work endometrial cells implant, proliferate, and invade the pelvic peritoneum. In endometriotic patients, genetic and epigenetic factors determine an impairment of these scavenger mechanisms, thus allowing endometrial cells to survive, proliferate, and implant into the peritoneum [21]. An increased activity of estrogen receptors (ERs), a local estrogen production in endometriotic cells, and a progesterone receptor decreased activity (progesterone-resistance) are considered the main causes of impaired apoptosis, reduced immune function, and increased inflammation [18]. When endometriotic cells invade the peritoneum, they grow and undergo cyclic tissue injury and repair, activating inflammatory cells (macrophages, T-cells) and the secretion of cytokines (interleukins). The inflammatory process also stimulates neoangiogenesis, neurogenesis, and fibrosis, which contribute explaining the pain symptoms [22].

Endometriosis is frequently linked to increased oxidative stress [23], which arises from a dysregulated balance between the production of reactive oxygen species (ROS) and the body’s antioxidant defense mechanisms [24]. ROS are byproducts of normal oxygen metabolism and act as inflammatory mediators that regulate cell proliferation but can also exert harmful effects [25]. In order to limit ROS production and mitigate potential damage, cells have developed a wide range of antioxidant systems, including superoxide dismutase, catalase, glutathione peroxidase, vitamin E, and vitamin C. In women with endometriosis, the imbalance between ROS production and antioxidant defense leads to oxidative stress [26], which promotes inflammation, contributing clinically to pain at affected lesions [24] and infertility [25].

### Endometriosis in Normal Weight and Obesity

Women with endometriosis show a higher prevalence of systemic comorbidities, related to a common endocrine, immune, and inflammatory background and to a high level of perceived stress [27]. Endometriosis and obesity are two common conditions that seem to be correlated; nevertheless, the underlying mechanisms are still uncertain [28]. Although obesity does not protect against endometriosis, an increased BMI may, in fact, lead to more severe disease stages [28]. This intricate relationship between obesity and severity of endometriosis appears to be underpinned by molecular mechanisms that see excessive adiposity-induced adipocyte dysfunction as a trigger for inflammation, oxidative stress, and angiogenesis. Together, these processes contribute to the exacerbation of the clinical manifestations of endometriosis [4].

Contrary to this, observational studies have consistently shown an inverse correlation between endometriosis and a low BMI [29, 30]. However, the observational nature of these studies makes this correlation debatable. Mechanistically, the correlation between low BMI and endometriosis may be explained by decreased intra-abdominal pressure, which could facilitate retrograde menstruation, a well-accepted initiator of endometriosis [31]. Another explanation may be related to behavioral aspects. Indeed, chronic pain and gastrointestinal disturbances (e.g., nausea and diarrhea) observed in patients with endometriosis can lead to appetite loss and subsequent caloric restriction [28]. Thus, the metabolic status of women should be considered in a broader context (e.g., diabetes, hypertension, dyslipidemia, and adiposity) rather than focusing solely on weight.

On the other hand, recent data highlighting the strong association between endometriosis and low BMI suggest a potential genetic Link. A 2021 study discussed the inverse correlation between endometriosis and low BMI, noting that this relationship may have a genetic background. The study emphasizes that while obesity does not protect against endometriosis, certain genetic polymorphisms are linked with the condition in females with varying BMI levels [28]. A Mendelian randomization analysis explored the genetic relationship between endometriosis and anthropometric traits, finding that reduced weight and BMI might mediate the genetic susceptibility to endometriosis, pointing toward a shared genetic origin for these traits [32]. Collectively, these studies underscore the potential genetic interplay between endometriosis and low BMI, highlighting the importance of considering genetic factors in understanding the etiology of endometriosis.

Endometriosis is a chronic disease requiring multidisciplinary management based on the patient’s symptoms and an individualized approach aiming to reduce pain, stress, and stress-related comorbidities and to improve quality of life. Dietary habits may influence endometriosis development and its management [5, 33]. However, it is important to bear in mind the difference between the impact that the diet could have on the risk of developing endometriosis and the effects of dietary interventions aimed at alleviating symptoms associated with the disease. Heterogeneous data addressing both perspectives can be found in the literature; nonetheless, data related to the association between nutrient intake and the risk of developing endometriosis remain non-causal [5].

### The Role of Hormones in Endometriosis

In patients with endometriosis serum levels of estradiol and progesterone are not significantly different from healthy women. The disease shows an estrogen dominance caused by an increased activity of ERs. A local synthesis of estradiol by endometriotic tissue is suggested by the evidence of increased aromatase activity, associated with an overexpression of cyclooxygenase 2 (COX2) and increased prostaglandins. In terms of alterations in the activity of ERs, an overexpression of ERβ and a downregulation of ERα have been observed in endometriosis [34].

Survival of endometriotic lesions, remodeling of peritoneal tissue, and production of pro-inflammatory substances are enhanced pathways triggered by ERβ. These changes stimulate nociceptors in pelvic tissues, causing pain and mediating immune system dysregulation in endometriotic lesions [35].

Endometriotic tissue is unable to respond to progesterone exposure, a phenomenon known as progesterone resistance. It manifests by the failure to induce transcription of progesterone-target genes despite adequate hormonal bioavailability [36]. Loss of progesterone responsiveness leads to the increased growth of endometriotic lesions and results in a non-receptive endometrium [31].

Anti-Mullerian hormone (AMH), a dimeric glycoprotein belonging to the transforming growth factor-β superfamily and a marker of ovarian reserve, shows significantly decreased serum levels in patients with OMA compared with age-matched fertile controls [37]. Moreover, the adverse effect of surgical removal of OMA on AMH levels is well recognized. Indeed, patients who have undergone a previous cystectomy present significantly lower serum AMH concentrations than those with OMAs who have never undergone surgery [38].

Patients with endometriosis show elevated stress levels. Infertility and pelvic pain cause increased anxiety and chronic stress [39]. Severe endometriosis-related pain (dysmenorrhea, pelvic pain, dyspareunia) usually presents with very high scores of perceived stress. Women undergoing multiple surgeries have reported high stress scores, indicating impairment of quality of life [40].

A stress-induced dysfunction of the hypothalamus-pituitary-adrenal axis is found in patients with endometriosis with an attenuated cortisol response, a condition known as burnout.

Autoimmune thyroid disorders are frequently observed in patients with endometriosis, a situation that suggests a physiopathological link between these two conditions [41]. Thyroid gland secretions can modulate immune responses, potentially contributing to the underlying mechanisms of endometriosis. Increased serum thyroid stimulating hormone (TSH) levels have been hypothesized as a participating factor for endometriosis development and progression. Patients with both endometriosis and thyroid disorders tend to experience more severe chronic pelvic pain and higher disease scores; hence, women with both conditions should be carefully monitored.

Since ovarian sex hormones influence the development of endometriosis, hormonal drugs targeting the hypothalamic-pituitary-ovarian axis are commonly used for treating patients with any form of endometriosis [42]. The main objective is to suppress menses, thus inducing iatrogenic menopause or pseudopregnancy, which in turn reduces pain symptoms in order to prevent or postpone surgery, which is useful for long-term management of the disease. First-line hormonal therapies include progestins [43], while second-line options are represented by gonadotropin-releasing hormone (GnRH) analogs or oral GnRH antagonists. Off-label use of combined oral contraceptives (COCs) is also common. New hormonal agents (aromatase inhibitors, selective estrogen receptor modulators [SERMs], and selective progesterone receptor modulators [SPRM]) are currently under investigation for the treatment of endometriosis. Hormonal therapies are the first choice, before surgery and after surgery, in order to reduce the risk of recurrence. The goal is to limit the negative effects of surgery on ovarian reserve and to manage endometriosis for a lifelong term by medical treatment [44]. Table 1 summarises the main hormonal alterations in endometriosis. Impaired regulation of activin A, contributes to inflammatory imbalance.

**Table 1:** Hormonal alterations in endometriosis and their clinical implications

Hormone	Change in Endometriosis	Clinical Implication
Estrogen (E2) [34]	↑ Local production	Promotes inflammation and supports lesion survival
Estrogen Receptor β (ERβ) [34]	↑ Expression	Enhances inflammatory signaling and cellular survival mechanisms.
Estrogen Receptor α (ERα) [34]	↓ Expression	Favors estrogen dominance, contributing to lesion persistence.
Progesterone (P4) [36]	↔ (Normal levels)	Ineffective counteraction of E2 due to receptor resistance.
Progesterone Receptors (PR-A, PR-B) [45]	↓ PR-B, ↓ PR-A (Resistance)	Associated with impaired progesterone signaling, lesion progression, and infertility.
Luteinizing Hormone (LH) [42]	↔ (Normal levels)	No significant differences in serum LH between women with endometriosis and healthy controls
Follicle-Stimulating Hormone (FSH) [42]		
Anti-Müllerian Hormone (AMH) [37].	↓ (especially in OMA and after surgery)	Marker of diminished ovarian reserve
Testosterone [46, 47]	↓ (generally lower androgen levels observed)	Endometriosis patients have significantly lower serum testosterone levels than women without endometriosis (Some studies found no statistically significant difference in total T. highlighting individual variability.)
DHEA-S (Dehydroepiandrosterone sulfate) [47]	↔ No significant change	In advanced endometriosis, mean levels of androstenedione, DHEA, andDHEA-S were slightly higher than in controls but without statistical significance (i.e., essentially no difference).
Activin A [48]	↑ in cystic fluid	Stimulates IL-6 and IL-8 expression, promoting local inflammation
Inhibin A/B [49]	↑ in cystic fluid	May facilitate ectopic endometrial tissue growth
Follistatin [50]	↓ Expression	Impaired regulation of Activin A, contributes to inflammatory imbalance.
Cortisol (salivary) [41]	↓ (blunted response), ↑ (in some hair/plasma studies)	Linked to dysregulated stress response and reduced endogenous analgesia
CRH / Urocortin [51]	↑ (especially in DIE lesions)	Promotes COX2 expression, enhancing inflammation, and pain sensitivity
TSH [52]	Normal to mildly ↑ (especially with thyroid autoimmunity)	Possible contribution to cell growth in endometrial tissue.
T3 [53]	↓ (especially with thyroid autoimmunity)	May impair stromal function and endometrial receptivity, contributing to subfertility.
T4 [54]	Normal to mildly ↑ (especially with thyroid autoimmunity)	Stimulates ROS, may increase ectopic cell proliferation, potentially linked to progesterone resistance and impaired implantation.

Anti-Müllerian Hormone, *AMH, *Cortisol Release Hormone, *CRH, *Deep Infiltrating Endometriosis, *DIE,* Estrogen, *E2,* Estrogen Receptor ,* ER*α, Estrogen Receptor β, *Er*β, Progesterone Receptor, *PR, *Thiroyd Stimulating Hormone, *TSH*

### Main Drug Therapies in Endometriosis

Drug therapies for the management of endometriosis aim at controlling symptoms and slowing the progression of the disease, especially considering the recurrence post-surgery [55]. With hormone suppression, the probability of lesion progression is not zero but is significantly lower than with expectant management [56]. Estroprogestins and COCs are consistently recommended by the majority of guidelines for the relief of related pain [57]. COCs can be used for long periods as a primary preventive measure, also potentially reducing the risk of ovarian cancer for women with endometriosis [58]. The mechanism through which COCs exert their therapeutic effects involves the suppression of ovarian activity, reducing the estrogen-induced production of prostaglandins and therefore tissue inflammation [59].

In addition, progestins alone are widely prescribed as first-line therapy [55]. Oral progestins are typically administered daily without interruption, leading to menstrual cycle cessation. This option could be of preference in women with metabolic or cardiovascular conditions that contraindicate the use of estroprogestins [60]. Dienogest is a fourth-generation oral progestin, used alone or in combination with estrogens for medical treatment of endometriosis [55]. It has demonstrated direct anti-inflammatory effects determining the reduction of inflammatory cytokines [55]. Moreover, intrauterine devices (IUD) releasing levonorgestrel are a very suitable option for cases with adenomyosis and increased risk for thrombotic conditions for whom systemic therapy could not be advisable [61, 62] (e.g., women above 40 years old, increased BMI, increased risk for estrogen-dependent diseases such as breast cancer or endometrial hyperplasia). The mechanisms through which progestins exert their therapeutic effects are the anti-estrogenic effects, anti-vasculogenic action, induction of apoptotic cascade in endometriotic cells, suppression of inflammatory pathways (as production of metalloproteinases), cell proliferation suppression, and also anti-neurogenic effects with inhibition of nerve growth within endometriotic lesions [59, 63, 64]. The question of whether a very low-dose COC used continuously or progestin monotherapy is better may be of little relevance, and it is plausible that ensuring amenorrhoea is more important than the modality chosen to achieve these goals [65].

GnRH agonists and antagonists are largely considered second-line treatment options when pain control is not achieved with first-line drugs [55]. These injectable drugs strongly suppress ovarian hormonal production, mainly estrogen, acting on the pituitary gland. In detail, GnRH antagonists rapidly inhibit gonadotropin release but without causing the flare-up effect that is associated with GnRH agonists' use [59]. Points against their use depend on the side effects due to the deep suppression of estrogen production, like hot flashes and bone loss, often needing the use of add-back therapy for long treatments (when more than 6 months long), consisting of supplementation of hormones to reduce these negative consequences [66]. Interestingly, dienogest was associated with an increased rate of spotting and weight gain, but alower rate of hot flashes and vaginal dryness compared to GnRH agonists [67]. Newly developed oral GnRH antagonists such as elagolix, relugolix, and linzagolix provide a useful option, avoiding the need for injection and allowing a prompt recovery of ovarian hormonal production upon discontinuation [59].

Furthermore, non-steroidal anti-inflammatory drugs (NSAIDs) are widely used medications against pelvic pain and dysmenorrhea, usually administered even before a definitive diagnosis. Their primary mechanism of action consists in the inhibition of the COX enzyme, which is essential for the synthesis of prostaglandins (PGs) [68]. Of note, endometriotic lesions have been observed expressing high concentrations of COX-2 receptors, suggesting a role for COX-2 inhibitors to reduce inflammation and pain due to endometriosis [69].

Another solution when previous options have proven insufficient for pain and disease control is represented by aromatase inhibitors, which locally suppress estrogen synthesis, inducing a hypoestrogenic state. [70, 71] They act by inhibiting the aromatase enzyme activity and blocking estrogen production both in the ovary and in the endometriotic lesions [70]. Since they also ameliorate folliculogenesis, a progestin, a COC, or a GnRH analogue should be added to ensure ovarian suppression. They can be a suitable option for postmenopausal women in whom surgery is contraindicated due to other medical conditions. However, routine clinical applications remain limited because of the potential adverse effects [70].

Indeed, if a woman with endometriosis expresses the desire to become pregnant, all these medical therapies cannot be taken into account, given the anti-estrogenic state caused by their administration. In this regard, ESHRE guidelines do not recommend any specific medical treatments to increase fertility [72], and ART treatments should be performed when needed, after a period of unprotected intercourse of at least 12 months, or 6 months above the age of 35 years old, failed to achieve pregnancy.

## ENDOMETRIOSIS: nutritionist’s Point of View

### Nutrition and Endometriosis

Although the pathophysiology of endometriosis is multifactorial, involving hormonal, immunological, genetic, and environmental contributors, growing attention has been given to the role of nutrition as both a modifiable risk factor and a potential adjunctive therapeutic approach [10]. Recent research supports the hypothesis that dietary patterns may influence the development, symptom severity, and progression of endometriosis through modulation of systemic inflammation, oxidative stress, and hormonal regulation [10, 23]. Adding to this growing body of evidence, a recent hospital-based case-control study investigated the relationship between the Alternative Healthy Eating Index (AHEI) and the likelihood of developing endometriosis [73]. The study, which included 105 women with endometriosis and 208 healthy controls, found that high adherence to the AHEI was associated with a 92% lower odds of endometriosis, even after adjusting for total energy intake, physical activity, reproductive history, and family history of the disease [73].

Several observational and clinical studies suggest that certain dietary components may exert protective or detrimental effects in women with endometriosis [74–79]. Diets rich in fruits, vegetables, whole grains, and omega-3 polyunsaturated fatty acids (PUFAs) have been associated with a reduced risk of endometriosis and improved symptom control [74–79]. Foods typically high in antioxidants and anti-inflammatory compounds, such as vitamins C and E, flavonoids, and carotenoids, could mitigate oxidative stress and modulate prostaglandin synthesis [80, 81], two processes implicated in the pathogenesis of endometriotic lesions [13]. Conversely, high consumption of red and processed meats, trans fats, and refined sugars has been correlated with increased endometriosis risk and exacerbation of symptoms [78, 82]. These foods promote a pro-inflammatory state and may increase estrogen levels or interfere with estrogen metabolism, potentially fostering the growth and maintenance of ectopic endometrial tissue [82]. The role of dietary fat composition in modulating inflammatory pathways is particularly noteworthy [83]. Specifically, trans fats and saturated fats,as well as excessive intake of omega-6 PUFAs, have been associated with the up-regulation of pro-inflammatory mediators involved in nociceptive signaling and the pathophysiology of endometriotic lesions. Conversely, monounsaturated fatty acids (MUFAs) and omega-3 PUFAs have been shown to inhibit the production of pro-inflammatory cytokines and may counteract the deleterious effects of omega-6 PUFAs, which are typically consumed in excess in Western dietary patterns. The omega-3 to omega-6 PUFA ratio has emerged as a key modulator of systemic and local inflammation [83], with implications for disease progression and symptomatology in endometriosis. Both preclinical and clinical studies suggest that omega-3 PUFA supplementation may lead to significant reductions in pelvic pain and lesion size, highlighting its potential as a therapeutic dietary strategy [84, 85].

Antioxidant supplementation has also gained attention as a supportive strategy [15, 86]. Vitamins C and E, NAC, and resveratrol have demonstrated efficacy in preclinical studies by reducing oxidative stress, modulating cytokine production, and impairing angiogenesis, all of which are key mechanisms in the development of endometriosis [15, 86]. A randomized controlled trial (RCT) demonstrated that daily supplementation with vitamins E and C significantly decreased pelvic pain and oxidative stress Markers in women with endometriosis over an 8-week period [87].

Additionally, the impact of dietary phytoestrogens—plant-derived compounds with estrogenic activity—has been debated [88]. Found in soy, flaxseed, and whole grains, phytoestrogens may either compete with endogenous estrogens for receptor binding, potentially exerting anti-estrogenic effects, or contribute to hormonal activity [88]. Current evidence is inconclusive, with some studies suggesting that phytoestrogens may help modulate hormonal balance and alleviate symptoms, while others raise concerns about their proliferative potential on estrogen-sensitive tissues [89–91].

Another promising area of investigation is the role of the gut microbiota in modulating the inflammatory and immunologic milieu associated with endometriosis [92]. Dysbiosis has been implicated in the disease’s progression, and dietary interventions aimed at restoring microbial diversity—such as high-fiber diets, prebiotics, and probiotics—are being explored for their therapeutic potential [92]. Some studies have reported improvements in pain and gastrointestinal symptoms among women with endometriosis following probiotic supplementation, suggesting a gut-immune axis involvement that may be amenable to nutritional modulation [93, 94]. In this context, the overlap between endometriosis and irritable bowel syndrome (IBS) has garnered increasing attention. Women with endometriosis are frequently misdiagnosed with IBS before a correct diagnosis is made, as both conditions share visceral hypersensitivity as a central feature. A retrospective analysis examined symptom profiles and dietary responses among women meeting Rome III criteria for IBS, comparing those with and without concurrent endometriosis [95]. The study found that dyspareunia, referred pain, menstruation-exacerbated bowel symptoms, and a family history of endometriosis were significantly associated with the co-occurrence of both conditions. Notably, a greater proportion of women with endometriosis and IBS experienced symptom improvement following a low FODMAP (fermentable oligosaccharides, disaccharides, monosaccharides, and polyols) diet compared to women with IBS alone [95]. This hypothesis has been supported by two recent interventional studies [96, 97]. In a randomized, controlled crossover feeding trial, 60% of women with endometriosis and gastrointestinal complaints responded to a low FODMAP diet compared to 26% on a control diet, with significant improvements in abdominal pain, bloating, stool form, and disease-related quality of life after four weeks (*p* < 0.001) [96]. Similarly, a second prospective study involving a structured low FODMAP protocol showed significant reductions in constipation, pain, and multiple domains of quality of life—particularly chronic pelvic pain, emotional well-being, and social functioning [97].

These findings support the hypothesis that targeted dietary interventions—such as the low FODMAP diet—may be particularly beneficial in specific subgroups of women.

While MNT cannot replace medical or surgical management of endometriosis, it represents a non-invasive, low-risk adjunctive strategy that may alleviate symptoms and improve quality of life. Nutritionists play a key role in translating emerging evidence into practical dietary interventions tailored to patients’ preferences and comorbidities. A holistic approach, integrating anti-inflammatory dietary principles, optimization of micronutrient intake, and support for gut health, may offer benefits beyond symptom control, including enhanced reproductive outcomes and reduced disease recurrence.

### Impact of the Mediterranean Diet on and Endometriosis

The MedDiet has been extensively studied and shown to offer various health benefits, including reducing the risk of cardiovascular disease (CVD), cancer, and type 2 diabetes [98–105]. This dietary pattern, rich in fruits, vegetables, legumes, and whole grains, is considered a potential non-pharmacological approach for endometriosis management. However, scientific evidence remains limited and inconsistent [106]. The connection between the MedDiet and endometriosis improvement is attributed to its anti-inflammatory properties, especially from fish and cold-pressed oils [107]. Extra virgin olive oil and fish, known for their anti-inflammatory effects, are key components of this diet [107–110]. Anti-inflammatory compounds in these foods, such as omega-3 PUFAs and squalene, a bioactive lipid, have demonstrated positive effects in the management of chronic diseases, including endometriosis [111]. Another key compound, oleocanthal, mimics the molecular structure of ibuprofen and inhibits cyclooxygenase through a similar mechanism [107, 109, 111, 112]. Recent research has specifically explored these compounds for their role in managing endometriosis symptoms [113]. In preclinical studies, Akyol et al. conducted a randomized, controlled, single-blind study in female rats and observed that omega-3 supplementation significantly reduced the size of endometriotic lesions [114]. In human studies, Nodler et al. reported decreased pelvic pain in women with endometriosis who supplemented with omega-3-rich fish oil, though similar results were observed in the placebo group [115].

Additionally, a higher intake of dietary fiber promotes digestion, while magnesium-rich foods may help regulate intracellular calcium levels, which are crucial for muscle contraction [107]. These dietary components may contribute to reducing chronic pelvic pain and systemic inflammation associated with endometriosis [107, 110, 112]. The persistent inflammation seen in endometriosis leads to the activation of nociceptors and both central and peripheral sensitization, contributing to chronic pain syndromes such as hyperalgesia and referred pain [116]. In women with endometriosis, pain is closely linked to tissue injury, inflammation, and oxidative stress [117]. While oxidative stress plays a signalling role in normal physiology, in endometriosis it contributes to pathological processes, including cell membrane damage, enzyme activation, DNA injury, and apoptosis [116].

The antioxidant-rich nature of the MedDiet, due to the consumption of fruits and vegetables, has been linked to a reduced risk of endometriosis, potentially due to the presence of beta-cryptoxanthin in these foods [107, 109, 118, 119].

Furthermore, common spices used in the MedDiet, such as onions, rosemary, chili peppers, ginger, turmeric, and garlic, have demonstrated anti-inflammatory properties [108]. Recent clinical studies support their effectiveness in preventing and alleviating various chronic conditions, including arthritis, asthma, cancer, neurodegenerative diseases, and cardiovascular disorders [9, 108, 120–126]. These spices may also help reduce inflammation associated with endometriosis [108].

Variations in the global prevalence of endometriosis suggest that, beyond genetic predisposition, environmental influences, lifestyle choices, and dietary patterns may also play a role in its development [127]. Implementing preventive strategies focused on modifying dietary habits may help alleviate gynecological complications and symptoms related to endometriosis [107, 128, 129]. Diet, being a modifiable factor, could impact both the progression and severity of endometriosis [127]. Nutritional factors may affect the disease through mechanisms such as alterations in estrogen and prostaglandin metabolism, modulation of smooth muscle activity, and regulation of inflammatory responses [130]. Estrogen plays a central role in stimulating the growth of endometrial cells, and certain dietary components can influence the body’s hormone metabolism and endogenous estrogen levels [113] (Table 2).

**Table 2:** Main studies on the effects of mediterranean diet on endometriosis

Study Type	Year	Number of patients	Intervention	Reference
Prospective study	2023	35	After 6 months on MD: assessed pain (VAS 0–10), vitamin levels, oxidative stress; found MD reduces pain in endometriosis	[116]
Cross-sectional	2020	113	Adherence to the MD was inversely related to the severity of menstrual pain	[134]
Experimental study	2012	68	5-month MD intervention in endometriosis led to pain relief and overall health improvement	[135]
Cross-sectional study	2023	505	Higher consumption of refined cereal, processed meats, and sugars was linked to more severe menstrual symptoms	[136]
Cross-sectional study	2024	105	Women with endometriosis reported lower consumption of fruits, vegetables, dairy products, and whole grains than recommended. Improving dietary quality could contribute to better well-being and reduced pain.	[137]
Case-control study	2021	156	High intake of fruits, red meat, yellow vegetables, dairy products, liquid oil, vegetables, potatoes, legumes, and low consumption of fried potatoes was related to a lower risk of endometriosis (*p* < 0.05). Increased risk observed with higher fried potato consumption	[138]
Retrospective case-control study	2019	156	A diet rich in fruits, vegetables, dairy products, fish, and meat was associated with reduced endometriosis symptoms.	[139]
Prospective cohort study	2022	3810	Higher carbohydrate quality and intake of total vegetable and cruciferous vegetable fiber were associated with lower risk of endometriosis diagnosis.	[140]
Case-control study	2020	207	Higher intake of green vegetables, red meat, dairy products, cheese, fresh fruits, and grain legumes was related to a lower risk of endometriosis.	[141]
Case-control study	2023	156	Women with endometriosis had lower intakes of key micronutrients (zinc, calcium, potassium, and phosphorus) compared to healthy controls.	[142]

Mediterranean Diet, MD; visual analogue scale, VAS

As knowledge of endometriosis progresses, new opportunities for innovative treatments will emerge, aiming to enhance the quality of life for individuals affected by the condition [131]. To advance research on the MedDiet and reproductive health, more trials are needed to establish specific nutritional recommendations.

Current studies often provide general MedDiet guidelines without addressing specific dietary components, and most research has been conducted in Western populations [132, 133]. Expanding research to diverse populations, both within and outside the Mediterranean region, is essential for a comprehensive understanding of the diet’s benefits [5, 107].

Standardizing MedDiet guidelines that define recommended food groups, portion sizes, and regional considerations is crucial. Consistency in study design, including uniform participant criteria, validated dietary assessment tools, duration, and control for potential confounders, is necessary. Implementing these recommendations will allow future research to generate more accurate and reliable findings about the relationship between the MedDiet and endometriosis [5, 107].

### Possible Impact of Ketogenic Diets in Endometriosis

Current therapeutic strategies for the treatment of endometriosis, such as hormonal suppression and surgical intervention, often yield only temporary relief and may involve notable side effects [143]. As a result, there is growing interest in exploring additional and alternative approaches, including dietary interventions [144]. Among these, KD—a high-fat, very low-carbohydrate, adequate-protein nutritional regimen—has garnered attention for its potent anti-inflammatory and metabolic benefits [145]. KD induces a metabolic shift from glucose to ketone bodies, especially β-hydroxybutyrate (BHB), as the primary energy substrate. BHB is known to inhibit the NLRP3 inflammasome and reduce the expression of key pro-inflammatory cytokines such as interleukin (IL)−1β, tumor necrosis factor (TNF)-α, and IL-6 [146, 147]. These mechanisms may be particularly beneficial in chronic inflammatory conditions like endometriosis, where immune dysregulation plays a central role in disease progression and symptom severity [148, 149]. Given that endometriosis is driven by chronic inflammation and oxidative stress—with elevated levels of peritoneal macrophages and pro-inflammatory cytokines [150]—the anti-inflammatory environment promoted by KD may help interrupt the pathophysiological cascade leading to lesion formation and pain. Additionally, disruptions in glucose and lipid metabolism have been implicated in the development of oxidative stress, endothelial dysfunction, and the activation of inflammatory signalling pathways [151]. KD’s ability to normalize blood glucose levels and enhance mitochondrial efficiency may further mitigate oxidative damage and improve cellular resilience.

Emerging evidence also points to the role of insulin resistance in endometriosis, especially in patients with overweight and obesity [152]. KD has demonstrated rapid improvements in insulin sensitivity and reductions in circulating insulin and glucose levels [153], suggesting that it could be particularly effective in metabolically vulnerable subgroups of endometriosis patients. Improving metabolic health may not only reduce systemic inflammation but also enhance the efficacy of conventional treatments by correcting underlying physiological alterations. Hormonal modulation remains a cornerstone of endometriosis management. Studies in patients with polycystic ovary syndrome (PCOS) have shown that KD can restore ovulatory cycles, reduce LH/FSH ratios, and lower androgen levels [154–156]. It is possible that similar effects could add benefit in individuals with endometriosis, especially those with comorbid reproductive or metabolic dysfunctions.

Pain, the hallmark symptom of the disease, stems not only from inflammation but also from neuroangiogenesis and central sensitization [157]. Preliminary observational studies and patient-reported outcomes suggest that KD may alleviate pain perception, potentially by modulating glutamatergic neurotransmission and reducing peripheral nociceptive input [158]. Such neuromodulator effects may contribute to long-term improvements in quality of life

Interestingly, a recent 12-week RCT provides additional clinical support for the use of KD in endometriosis [159]. In this study, women with endometriosis were randomized to receive either standard treatment alone or in combination with a medium-chain triglyceride (MCT)-modified KD. The intervention group experienced significant reductions in dyspareunia and dyschezia compared to controls, with a marginally significant reduction in pelvic pain. Although no significant changes in anthropometric or biochemical parameters were observed, these findings highlight the potential symptom-relieving effects of KD, even in the absence of weight loss or major metabolic shifts [159]

In consideration of the evidence linking red and processed meat consumption with increased endometriosis risk [160], a Mediterranean Ketogenic Diet—focusing on fish, eggs, white meat, plant-derived fats, and fiber-rich vegetables, excluding red and processed meats—may offer a new synergistic nutritional option. This approach combines the anti-inflammatory, metabolic, and hormonal benefits of KD with the vascular-protective and antioxidant-rich properties of the Mediterranean diet [161, 162], potentially offering a more sustainable and food-based therapeutic option for managing endometriosis. Further RCTs are warranted to validate these promising dietary strategies and elucidate their mechanisms in the context of endometriosis.

### Dietary Supplements for Treatment of Endometriosis

The complex physiopathology of endometriosis often requires conventional medical and surgical therapies; however, dietary supplements may offer synergistic therapeutic effects due to their immunomodulatory, anti-inflammatory, antioxidant, and antiproliferative properties [16, 117, 119, 124, 163–168]. Dietary supplements include vitamins, lipids, and trace elements, which have been investigated for their potential to modulate immune responses and oxidative stress in endometriosis [169–174].

A recent large-scale cross-sectional study further underscored the relevance of dietary interventions in the self-management of endometriosis-associated pain [175]. A recent 24-question online survey conducted in collaboration with a local patient support group, and published collected responses from 2388 individuals with confirmed endometriosis (mean age 35.4 years, 88.4% White). Among them, 83.8% reported having tried at least one dietary strategy, and 58.8% had used dietary supplements to Manage symptoms. Of these, 66.9% and 43.4%, respectively, perceived an improvement in pain levels. The majority also reported pelvic pain (96.9%) and frequent abdominal bloating (91.2%), highlighting the burden of gastrointestinal comorbidities, colloquially referred to as “endo belly.” Although not interventional, this study reinforces the strong interest in diet and supplement-based self-care among individuals with endometriosis and suggests that dietary modification may be perceived as beneficial by patients despite limited clinical guidance [175].

Vitamin D, or 1,25-dihydroxyvitamin-D3, modulates the immune system and influences cellular differentiation and proliferation [16, 176–178]. Notably, vitamin D receptors and metabolizing enzymes are present in the ovaries and endometrium of women with and without endometriosis [16], suggesting a local immunomodulatory role. Emerging evidence indicates that vitamin D may contribute to the management of chronic inflammatory and autoimmune conditions by enhancing anti-inflammatory cytokines,such as transforming growth factor-beta (TGF-β) and IL-4,while down-regulating pro-inflammatory mediators, including TNF-α, IL-2, and IL-6 [16].

Multiple studies have demonstrated an inverse correlation between serum vitamin D levels and endometriosis risk [179–182]. Women with lower circulating levels of vitamin D are more likely to develop endometriosis, and deficiency may be associated with increased disease severity [179, 180, 183]. Based on this data, the potential therapeutic benefits of vitamin D supplementation in endometriosis have been explored, yielding mixed results. An in vitro study showed that vitamin D reduced inflammatory markers, including IL-8 expression and prostaglandin activity [184]. Also, a RCT comparing vitamin D, fish oil, and placebo suggested that vitamin D might alleviate endometriosis-related pain; however, no significant difference was observed compared to placebo [115].

A recent systematic review of registered RCTs evaluated the effects of vitamin D supplementation on pain outcomes in individuals with primary dysmenorrhea or endometriosis [185]. Among the seven included trials, considerable heterogeneity in dosage, duration, and methodological quality was observed. While vitamin D significantly reduced pain severity in cases of primary dysmenorrhea (mean difference − 1.12, 95% CI − 2.16 to − 0.07), no significant effect was found in endometriosis-related RCTs. These findings suggest a potential role for vitamin D in dysmenorrhea management, but limited evidence supports its efficacy for endometriosis-associated pain [185].

Furthermore, oxidative stress and inflammation are also well-documented contributors to endometriosis pathophysiology [186, 187]. Affected patients frequently exhibit reduced antioxidant levels in peritoneal fluid alongside elevated inflammatory markers such as IL-1, IL-6, monocyte chemoattractant protein-1 (MCP-1), and TNF-α [186]. In this context, antioxidant vitamins have garnered interest. An RCT evaluating combined vitamin C and E supplementation demonstrated reductions in chronic pelvic pain, dysmenorrhea, and dyspareunia, alongside decreased inflammatory markers in peritoneal fluid [186]. Another RCT found that supplementation with vitamins C and E significantly reduced levels of malondialdehyde (MDA) and lipid hydroperoxides (LOOHs) after 4 and 6 months of treatment, though no improvements in pregnancy rates were reported [188]. Finally, in another RCT, daily vitamin E and C supplementation for 8 weeks reduced serum ROS and MDA, and also improved the severity of pelvic pain, dysmenorrhea, and dyspareunia [87].

Omega-3 fatty acids, a subclass of PUFAs, have demonstrated antiproliferative, antiangiogenic, anti-inflammatory, and anti-apoptotic effects in several studies [189]. However, one early study found that omega-3 supplementation did not significantly improve pain scores or quality of life in women with endometriosis [115].

One powerful anti-inflammatory is curcumin [190]. Numerous studies have demonstrated curcumin’s antioxidant, anti-inflammatory, and antiangiogenic qualities [190]. In an RCT involving women with endometriosis, 1000 mg of curcumin administered daily for eight weeks did not result in significant improvements in pain or quality of life compared to placebo [191]

Other dietary supplements have been studied, like quercetin [16]. It is a flavonol found in vegetables and fruits, which inhibits proliferation and induces cell cycle arrest in endometriotic cells in vitro [16]. Although limited and low-quality studies have assessed its clinical effects, often in combination with other supplements like linoleic acid, linolenic acid, and nicotinamide, some evidence suggests a reduction in pain and serum PG-E2 levels [192].

Recently, two meta-analyses further investigated the efficacy of dietary supplements for managing endometriosis-associated pain. One meta-analysis, including nine RCTs, found no significant difference between supplements and placebo in improving pelvic pain, dysmenorrhea, or dyspareunia, and highlighted the overall lack of high-quality evidence supporting supplement use for symptom relief [193]. In contrast, a second systematic review and meta-analysis reported that anti-inflammatory dietary supplements significantly reduced pelvic pain, particularly in patients older than 32 years with stage III–IV disease, BMI > 23 kg/m², and supplement durations longer than eight weeks [194].

In summary, nutritional interventions with targeted micronutrients appear to offer potential as adjunctive therapies in endometriosis management. Vitamin D supplementation has shown beneficial effects on pain in some studies, while antioxidant vitamins C and E may reduce pain, inflammation, and oxidative stress. Although compounds such as omega-3 fatty acids, curcumin, and quercetin show promise, recent evidence underscores the need for further high-quality trials before recommending these supplements in clinical practice. In particular, further evidence is needed to establish the effective role of nutraceutical supplementation in combination with specific, targeted dietary interventions, as current studies have not investigated this potential synergistic effect. An important limitation of the available RCTs is the lack of standardization in supplement dosing and the variable bioavailability of these compounds, which May contribute to the heterogeneity of clinical outcomes. A summary of all studies is expressed in Table 3.

**Table 3:** Summary of randomised clinical trials investigating the effects of vitamin and phytochemical supplementation on endometriosis-related symptom

Dietary Supplement	Type of Study/Intervention	Effect	Reference
Vitamin D3 or Fish Oil	RCT: 147 women aged 12-25, received 2,000 IU of vitamin D3, or 1000 mg fish oil or placebo for 6 months	Vitamin D3 supplementation improved VAS pain scores.	[115]
Vitamin D	RCT: 39 women diagnosed after laparoscopy, received 50,000 IU of vitamin D weekly or placebo for 12 weeks	Vitamin D did not improve pain severity or dysmenorrhea	[195]
Vitamin D	RCT: 60 women aged 18-40, received 50,000 IU of vitamin D every 2 weeks for 12 weeks or placebo	Vitamin D decreased pelvic pain, high-sensitivity C-reactive protein, and increased total antioxidant capacity	[196]
Vitamin C and E	RCT: 59 women aged 19-41, received 1,200 IU of vitamin E and 1000 mg of vitamin C daily for 8 weeks or placebo	Treatment reduced daily pain, dysmenorrhea, dyspareunia, and IL-6 and MCP-1 in peritoneal fluid	[186]
Vitamin C and E	RCT: 34 women, received 343 mg of vitamin C and 84 mg of vitamin E daily for 6 months or placebo	Treatment reduced plasma LOOHs and MDA concentration at 4 and 6 months, no difference in pregnancy rate	[188]
Vitamin C and E	RCT: 60 women aged 15-45, received 1,000 mg of vitamin C and 800 IU of vitamin E for 8 weeks or placebo	Treatment reduced serum ROS and MDA levels, reduced pelvic pain, dysmenorrhea, and dyspareunia	[87]
Curcumin	RCT: 68 women received 1,000 mg of curcumin for 8 weeks or placebo.	No differences in pain or quality of life	[191]

lipid hydriperoxides, LOOHs; Malondialdehyde, MDA; Randomized Controlled Trial, RCT; Visual Analog Scale, VAS; International Units, IU

## Conclusions

Endometriosis is a complex, estrogen-dependent, chronic inflammatory disease characterized by the ectopic presence of endometrial-like tissue outside the uterine cavity. This disease is frequently associated with dysmenorrhea, chronic pelvic pain, infertility, and systemic inflammation. Given the multifactorial etiology of its pathophysiology, which includes hormonal, metabolic, and immunological factors, MNT has proven to be a valid non-pharmacological therapeutic tool in the integrated management of this complex disease. Indeed, in addition to pharmacological and surgical therapy, nutritional interventions represent a promising therapeutic strategy to manage symptoms and reduce disease progression.

**Fig. 1:**
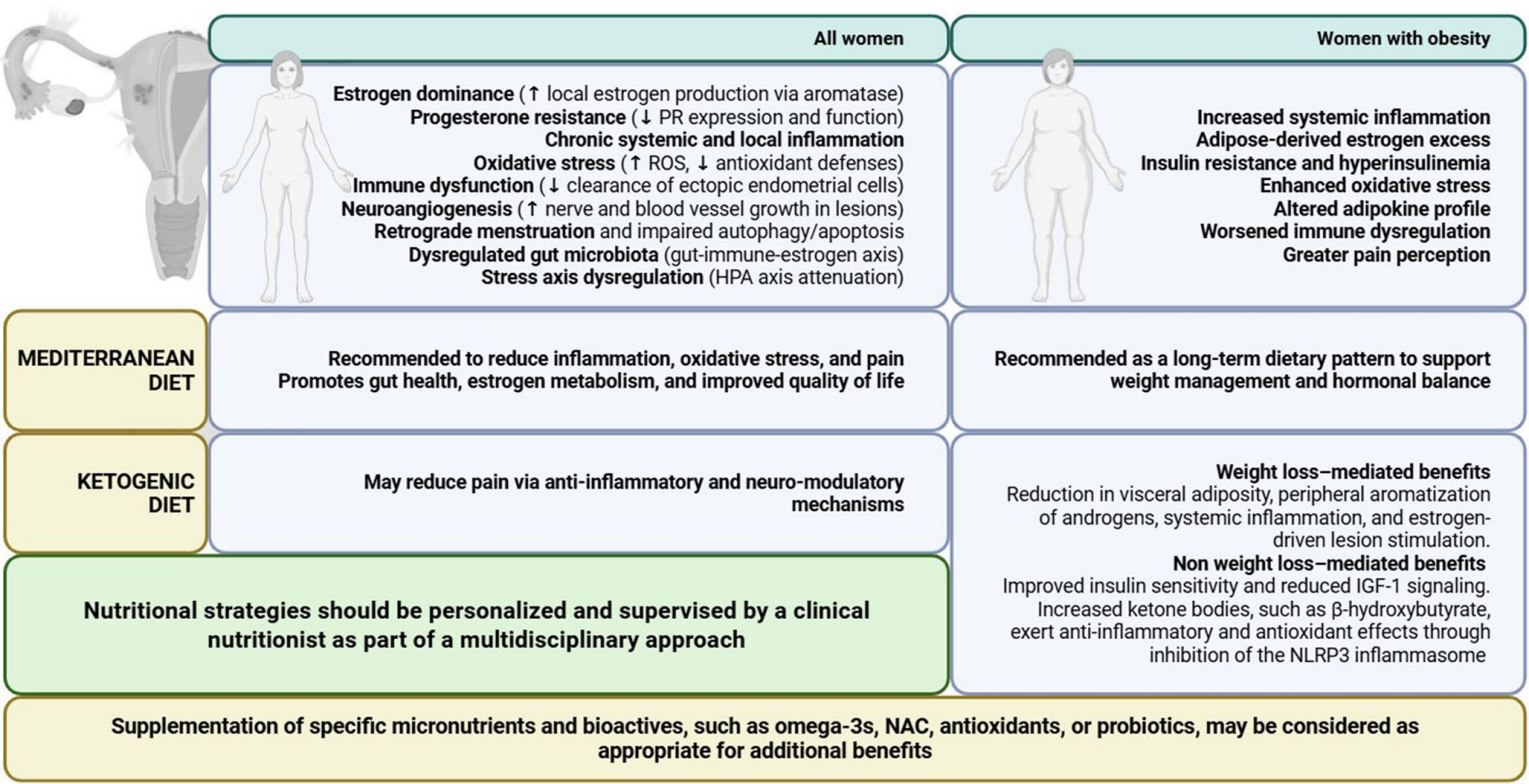
Medical nutrition therapy (MNT) plays a crucial role in the complex clinical management of endometriosis. The Mediterranean diet should always be considered in the phenotype of patients with normal weight endometriosis, while the ketogenic diet can be evaluated as a short-term strategic tool in patients with endometriosis and obesity, especially when there are endocrine and metabolic dysfunctions that require rapid action on improving metabolism and reducing inflammation. The clinical nutritionist therefore plays a fundamental role in the evaluation and monitoring in the choice of the best-personalized MNT for the patient and the possible use of specific nutraceuticals, ensuring an integration based on medical-nutritional evidence, safety and effective in the nutritional and multidisciplinary management of endometriosis.

Among the most frequently used dietary therapies in clinical practice, we find the MedDiet and KD, each offering distinct mechanistic advantages. The prescription of the most appropriate dietary approach must be personalized according to the clinical phenotype of the individual patient (Fig. 1). The prescription of the specific MNT must be individualized based on the patient’s symptom profile, metabolic status, body weight, and reproductive goals.

Furthermore, the use of nutraceuticals could enhance the effects of dietary interventions, offering additional therapeutic benefits.

The MedDiet, characterized by a high intake of whole grains, legumes, extra virgin olive oil, fish, vegetables, and fruits, with moderate consumption of dairy and red wine, is rich in monounsaturated fats, polyphenols, fiber, and omega-3 fatty acids. This dietary pattern exerts significant anti-inflammatory and antioxidant effects, reducing systemic chronic inflammation through modulation of cytokines production (e.g., IL-6, TNF-α) and reduction of oxidative stress. Furthermore, Mediterranean-type dietary therapy improves hormonal balance and estrogen metabolism through its beneficial impact on the modulation of the intestinal microbiota and hepatic metabolism. In normal-weight women with endometriosis, especially those with chronic pelvic pain, gastrointestinal symptoms, or infertility, the MedDiet can represent an optimal long-term nutritional strategy. Its benefits extend to improved oocyte quality and endometrial receptivity. The MedDiet also contributes to improved psychological well-being, which is often compromised in this patient population.

In contrast, KD, defined by high-fat, very low-carbohydrate, and moderate protein intake, induces a state of nutritional ketosis with elevated circulating ketone bodies, including β-hydroxybutyrate. These compounds have demonstrated analgesic, anti-inflammatory, and antioxidants properties through inhibition of the NLRP3 inflammasome and modulation of pain signaling pathways. The KD may be especially beneficial in women with obesity and endometriosis, who often present with an altered metabolic phenotype with insulin resistance, chronic inflammation, and increased peripheral aromatization of androgens into estrogens in adipose tissue by aromatases. Weight loss, especially visceral weight loss, induced by the ketogenic diet can reduce estrogen levels by reducing systemic inflammation and hepatic metabolism, potentially also inhibiting the progression of endometriosis. However, due to the controlled energy and macronutrient intake, as well as the potential long-term adverse effects, the ketogenic diet should be used for limited periods, possibly cycled, under the strict supervision of a nutritionist.

Finally, nutraceutical supplementation could further improve the efficacy of MNT. Compounds such as resveratrol, curcumin, NAC), omega-3 fatty acids, and quercetin have the potential to inhibit angiogenesis, reduce oxidative damage, modulate inflammatory pathways, and attenuate pain. Their use may be particularly beneficial in patients with persistent symptoms despite dietary and lifestyle interventions or in combination with pharmacological therapy.

In conclusion, MNT plays a crucial role in the complex clinical management of endometriosis. The MedDiet should always be considered in the phenotype of patients with normal weight endometriosis, while the KD can be evaluated as a short-term strategic tool in patients with endometriosis and obesity, especially when there are endocrine and metabolic dysfunctions that require rapid action on improving metabolism and reducing inflammation. The clinical nutritionist therefore plays a fundamental role in the evaluation and monitoring in the choice of the best-personalized MNT for the patient and the possible use of specific nutraceuticals, ensuring an integration based on medical-nutritional evidence, safety and effective in the nutritional and multidisciplinary management of endometriosis

Nutrition represents a promising frontier in the comprehensive management of endometriosis. Future research should focus on longitudinal and interventional studies to better define the optimal dietary patterns and nutrient supplementation protocols. Precision nutrition approaches, considering genetic, microbiome, and metabolic profiles, may further refine individualized dietary strategies for women affected by this complex and often debilitating condition

 Hormonal alterations in endometriosis and their clinical implications.

## Key References

- Taylor, H. S., Kotlyar, A. M., & Flores VA. Endometriosis is a chronic systemic disease: clinical challenges and novel innovations. Lancet (London, England). 2021;397:839–52.
  - Endometriosis is now recognized as a systemic disease that extends beyond the pelvis, affecting metabolism, inflammation, and the central nervous system
- Arab, A., Karimi, E., Vingrys, K., Kelishadi, M. R., Mehrabani, S., & Askari G. Food groups and nutrients consumption and risk of endometriosis: a systematic review and meta-analysis of observational studies. Nutr J. 2022;21:58.
  - Higher consumption of fruits, vegetables, and dairy products may reduce the risk of endometriosis, while high intake of red meat and trans fats appears to increase it
- Sverrisdóttir, U. Á., Hansen, S., & Rudnicki M. Impact of diet on pain perception in women with endometriosis: A systematic review. Eur J Obstet Gynecol Reprod Biol. 2022;271:245–9.
  - Dietary interventions may influence pain perception in women with endometriosis, highlighting the potential of nutrition as part of pain management strategies.
- Rashidian, P., Amini-Salehi, E., Karami, S., Nezhat, C., & Nezhat F. Exploring the Association Between Dietary Fruit Intake and Endometriosis: A Systematic Review and Meta-Analysis. J Clin Med. 2025;14:1246.
  - Greater fruit intake is inversely associated with the risk of endometriosis, supporting a protective role of fruit-rich diets.
- Field, R., Pourkazemi, F., & Rooney K. Effects of a Low-Carbohydrate Ketogenic Diet on Reported Pain, Blood Biomarkers and Quality of Life in Patients with Chronic Pain: A Pilot Randomized Clinical Trial. Pain Med. 2022;23:326–38.
  - A well-formulated ketogenic diet led to greater reductions in pain, inflammation, weight, and mood symptoms compared to a whole-food diet alone in patients with chronic musculoskeletal pain

## Supplementary Information

Below is the link to the electronic supplementary material.

ESM 1 (PDF 1.41 MB)

## Data Availability

No datasets were generated or analysed during the current study.
