# Supplementary material for: Effectiveness of Medical Nutrition Therapy in the Management of Patients with Obesity and Endometriosis: from the Mediterranean Diet To the Ketogenic Diet, Through Supplementation. The Role of the Nutritionist in Clinical Management
Source: Curr Obes Rep. 2025 Sep 8;14(1):68. doi: 10.1007/s13679-025-00662-8 (PMC12417264; doi:10.1007/s13679-025-00662-8)
Supplement: Supplementary file 1 — (PDF 1.41 MB) [file 13679_2025_662_MOESM1_ESM.pdf]

SPRINGER NATURE

Change of authorship request form - Journals (pre-acceptance)

Section 1: Please provide the current title of manuscript

Manuscript ID no.: 0576a9a9-f499-4f4e-b1ea-340179afcb62 v2.0

Title: Effectiveness of Medical Nutrition Therapy in the Management of Patients with Obesity and Endometriosis: From the Mediterranean diet to the Ketogenic diet, through supplementation. The Role of the Nutritionist in Clinical Management

Section 2: Please provide the previous authorship, in the order shown on the manuscript before the changes were introduced. Please indicate the corresponding author by adding (CA) behind the name.

	First name(s)	Family name	ORCID or SCOPUS id, if available
1st author	Luigi	Barrea	Scopus ID: 55544325400; ORCID: 0000-0001-9054-456X
2nd author	Ludovica	Verde	Scopus ID: 57303571200; ORCID: 0000-0002-4583-8283
3rd author	Giuseppe	Annunziata	Scopus ID: 57208131064; ORCID: 0000-0002-1922-662X
4th author	Peter	Chedraui	Scopus ID: 6601957219
5th author	Felice	Petraglia	Scopus ID: 55075291000 ORICD: 0000-0002-8851-625X
6th author	Gabriela	Cucalón	Scopus ID: 57724893200 ORCID: 0000-0002-6900-5649
7th author	Elisabetta	Camajani	Scopus ID: 57209796548
8th author	Massimiliano	Caprio	Scopus ID: 58169767000; ORCID: 0000-0003-0722-7163
9th author	Stefania	Gorini	Scopus ID: 24597321900
10th author	Luigi	Carbone	Scopus ID: 57203899419; ORCID: 0000-0002-2127-1876
11th author	Sebastian	Chapela	Scopus ID: 23501307600; ORCID: 0000-0002-8083-1714
12th author	Evelyn	Frias-Toral	Scopus ID: 57218208930; ORCID:0000-0002-2228-0141
13th author	Giovanna (CA)	Muscogiuri	Scopus ID: 25636000100; ORCID: 0000-0002-8809-4931

SPRINGER NATURE Springer Nature is one of the world's leading global research, educational and professional publishers, created in May 2015 through the combination of Nature Publishing Group, Palgrave Macmillan, Macmillan Education and Springer Science+Business Media.

SPRINGER NATURE

Change of authorship request form - Journals (pre-acceptance)

Important information. Please read.

- This form should be used by authors to request any change in authorship (adding/deleting authors) including changes in corresponding authors. This form should not be used for name changes. Please fully complete all sections. Use black ink and block capitals and provide each author's full name with the given name first followed by the family name.

- By signing this declaration, all authors guarantee that the order of the authors are in accordance with their scientific contribution, if applicable as different conventions apply per discipline, and that only authors have been added who made a meaningful contribution to the work.

- Please note, in author collaborations where there is formal agreement for representing the collaboration, it is sufficient for the representative or legal guarantor (usually the corresponding author) to complete and sign the Authorship Change Form on behalf of all authors, **next to the added/removed author(s). (Complete Section 3, followed by Section 6.)**
  In author collaborations where there is no formal agreement for representing the collaboration and **there are more than 10 authors**, one may sign for all, provided the signer appends correspondence that attests that each of the authors have agreed to the change **and the added/removed authors sign the form. (Complete Section 3, followed by Section 6.)**

- Please note, we cannot investigate or mediate any authorship disputes. If you are unable to obtain agreement from all authors (including those who you wish to be removed) you must refer the matter to your institution(s) for investigation. Please inform us if you need to do this.

- If you are not able to return a fully completed form within **30 days** of the date that it was sent to the author requesting the change, we may have to withdraw your manuscript. We cannot publish manuscripts where authorship has not been agreed by all authors (including those who have been removed).

- Incomplete forms will be rejected.

- Please return/upload this form, fully completed, to the Journals Editorial Office. The Journal and/or Publisher will consider the information you have provided to decide whether to approve the proposed change in authorship. We may decide to contact your institution for more information or undertake a further investigation, if appropriate, before making a final decision.

SPRINGER NATURE Springer Nature is one of the world's leading global research, educational and professional publishers, created in May 2015 through the combination of Nature Publishing Group, Palgrave Macmillan, Macmillan Education and Springer Science+Business Media.

SPRINGER NATURE

Change of authorship request form - Journals (pre-acceptance)

Section 3: Please provide a justification for change. Please use this section to explain your reasons for changing the authorship of your manuscript, e.g. what necessitated the change in authorship? Please refer to the (journal) policy pages for more information about authorship. Please explain why omitted authors were not originally included and/or why authors were removed on the submitted manuscript.

We would like to request a revision of the authorship list for our manuscript to include two collaborators who contributed significantly to the study from its inception. Unfortunately, due to an oversight during the initial submission, their names were unintentionally omitted. Both collaborators were involved from the early stages of the research and manuscript drafting. Their contributions align with the journal's criteria for authorship, and we sincerely apologize for the omission. We respectfully request your understanding in allowing us to correct this error and update the author list accordingly.

Section 4: Proposed new authorship. Please provide your new authorship list in the order you would like it to appear on the manuscript. Please indicate the corresponding author by adding (CA) behind the name. If the Corresponding Author has changed, please indicate the reason under section 3.

	First name(s)	Family name (this name will appear in full on the final publication and will be searchable in various abstract and indexing databases)	Affiliated institute	E-mail address
1st author	Luigi	Barrea	Università Telematica Pegaso, Naples, Italy	luigi.barrea@unipegaso.it
2nd author	Ludovica	Verde	University of Naples Federicó II, Naples, Italy	ludovica.verde@unina.it
3rd author	Giuseppe	Annunziata	Università Telematica Pegaso, Naples, Italy	giuseppe.annunziata@unipegaso.it
4th author	Peter	Chedraui	Universidad Espíritu Santo, Samborondón, Ecuador	peterchedraui@hotmail.com
5th author	Felice	Petraglia	University of Florence, Careggi University Hospital, Florence, Italy	felice.petraglia@unifi.it
6th author	Gabriela	Cucalón	ESPOL Polytechnic University, Guayaquil, Ecuador	mcucalon@espol.edu.ec
7th author	Elisabetta	Camajani	IRCCS San Raffaele, Rome, Italy	elisabetta.camajani@uniroma5.it
8th author	Massimiliano	Caprio	IRCCS San Raffaele, Rome, Italy	massimiliano.caprio@sanraffaele.it
9th author	Stefania	Gorini	San Raffaele Open University, Rome, Italy	stefania.gorini@uniroma5.it
10th author	Giuseppe Gabriele	Iorio	University of Naples Federico II, Naples, Italy	giuseppeg.iorio94@gmail.com

SPRINGER NATURE Springer Nature is one of the world's leading global research, educational and professional publishers, created in May 2015 through the combination of Nature Publishing Group, Palgrave Macmillan, Macmillan Education and Springer Science+Business Media.

11th author	Raffaella	Di Girolamo	University of Naples Federico II, Naples, Italy	raffaella.digirolamo95@gmail.com
12th author	Luigi	Carbone	University of Naples Federico II, Naples, Italy	drcarboneluigi@gmail.com
13th author	Sebastian	Chapela	Universidad de Buenos Aires, Ciudad Autónoma de Buenos Aires, Argentina	sebachapela@gmail.com
14th author	Evelyn	Frias-Toral	Universidad Católica de Santiago de Guayaquil, Guayaquil, Ecuador	evelyn.frias@cu.ucsg.edu.ec
15th author	Giovanna (CA)	Muscogiuri	University of Naples Federico II, Naples, Italy	evelyn.frias@cu.ucsg.edu.ec

Please use an additional sheet if there are more than 10 authors.

SPRINGER NATURE Springer Nature is one of the world's leading global research, educational and professional publishers, created in May 2015 through the combination of Nature Publishing Group, Palgrave Macmillan, Macmillan Education and Springer Science+Business Media.

SPRINGER NATURE

Change of authorship request form - Journals (pre-acceptance)

Section 5: Author contribution, Acknowledgement and Disclosures. Please use this section to provide a new disclosure statement and, if appropriate, acknowledge any contributors who have been removed as authors and ensure you state what contribution any new authors made (if applicable per the journal or book (series) policy). Please ensure these are updated in your manuscript - after approval of the change(s) - as our production department will not transfer the information in this form to your manuscript.

New acknowledgements:
Not applicable

New Disclosures (financial and non-financial interests, funding):
Competing Interests
The authors have no competing interests to declare that are relevant to the content of this article.
Funding
The authors did not receive support from any organization for the submitted work.

New Author Contributions statement (if applicable per the journal policy):
Conceptualization: GM and LB; Literature search: all; Original draft preparation: all, Writing - review and editing: L.V. and G.A; Supervision: GM and LB.

SPRINGER NATURE Springer Nature is one of the world's leading global research, educational and professional publishers, created in May 2015 through the combination of Nature Publishing Group, Palgrave Macmillan, Macmillan Education and Springer Science+Business Media.
